# Platelets in Non-alcoholic Fatty Liver Disease

**DOI:** 10.3389/fphar.2022.842636

**Published:** 2022-02-18

**Authors:** Andrea Dalbeni, Marco Castelli, Mirko Zoncapè, Pietro Minuz, David Sacerdoti

**Affiliations:** ^1^ Division of General Medicine C, Department of Medicine, University and Azienda Ospedaliera Universitaria Integrata of Verona, Verona, Italy; ^2^ Liver Unit, Department of Medicine, University and Azienda Ospedaliera Universitaria Integrata of Verona, Verona, Italy

**Keywords:** non-alcoholic fatty liver disease, non-alcoholic steatohepatitis, inflammation, fibrosis, platelets, Kupffer cells, hepatic stellate cells, antiplatelet agents

## Abstract

Non alcoholic steatohepatitis (NASH) is the inflammatory reaction of the liver to excessive accumulation of lipids in the hepatocytes. NASH can progress to cirrhosis and hepatocellular carcinoma (HCC). Fatty liver is the hepatic manifestation of metabolic syndrome. A subclinical inflammatory state is present in patients with metabolic alterations like insulin resistance, type-2 diabetes, obesity, hyperlipidemia, and hypertension. Platelets participate in immune cells recruitment and cytokines-induced liver damage. It is hypothesized that lipid toxicity cause accumulation of platelets in the liver, platelet adhesion and activation, which primes the immunoinflammatory reaction and activation of stellate cells. Recent data suggest that antiplatelet drugs may interrupt this cascade and prevent/improve NASH. They may also improve some metabolic alterations. The pathophysiology of inflammatory liver disease and the implication of platelets are discussed in details.

## Introduction

The term non-alcoholic fatty liver disease (NAFLD) was coined by Ludwig and colleagues (Ludwig et al., 1980) to describe fatty liver disease arising in the absence of significant alcohol intake.

NAFLD is histologically characterized by macrovesicular steatosis and further categorized into non-alcoholic fatty liver (NAFL) and non-alcoholic steatohepatitis (NASH) a more severe and evolutive disease, including inflammation and balooning.

The definition by EASL Guidelines for the management of non-alcoholic fatty liver disease is the following: NAFLD characteristic is the excessive hepatic fat accumulation, which is associated with insulin resistance. NAFLD is also defined by the presence of steatosis in more than 5% of hepatocytes in histological analysis or by density fat fraction exceeding 5.6% as assessed by proton magnetic resonance spectroscopy or quantitative fat/water selective magnetic resonance imaging. The term NAFLD includes two distinct conditions with different prognosis: non-alcoholic fatty liver (NAFL) and non-alcoholic steatohepatitis (NASH). While NAFL is a milder condition, NASH covers a wide spectrum of disease severity, including fibrosis, cirrhosis and hepatocellular carcinoma (HCC). (Lazarus et al., 2021).

Now the term metabolic associated fatty liver disease (MAFLD) has been adopted, instead of NAFLD/NASH, chosen by a group of experts (Eslam et al., 2020). Three criteria define MAFLD when associated with accumulation of fat in the liver, when diagnosed by histology, imaging, or blood biomarkers: 1) overweight/obesity, 2) type 2 diabetes mellitus, or 3) metabolic dysregulation consisting of at least two of the following risk conditions: waist circumference ≥102/88 cm in Caucasian men and women or ≥90/80 cm in Asian men and women, blood pressure ≥130/85 mmHg or antihypertensive treatment, plasma triglycerides ≥150 mg/dl or specific drug treatment plasma, HDL-cholesterol <40 mg/dl in men and <50 mg/dl in women or specific drug treatment, fasting glucose levels 100–125 mg/dl, or 2-h post-load glucose levels 140–199 mg/dl or HbA1c 5.7–6.4%, insulin resistance score ≥2.5. Plasma high-sensitivity C-reactive protein level >2 mg/L.

Another condition, MAFLD-related cirrhosis, is characterized by cirrhosis with past or present evidence of metabolic risk factors that meet the criteria to diagnose MAFLD, with at least one of the following: 1) Documentation of MAFLD on a previous liver biopsy. 2) Historical documentation of steatosis by hepatic imaging (Eslam et al., 2020). Being a new entity, few papers are available on MAFLD, and this review will refer only to NAFLD/NASH.

## Epidemiology of Non-Alcoholic Fatty Liver Disease

Risk factors for the development of NAFLD, or its evolution to NASH, are the same of metabolic syndrome, a disorder consisting by definition of obesity, arterial hypertension, impaired glucose metabolism and atherogenic dyslipidaemia, a clinical condition with high prevalence in the adult population worldwide, particularly in industrialized countries (Younossi et al., 2019).

In some countries, NAFLD represents the primary cause of cirrhosis (Moon et al., 2020; Setiawan et al., 2016), the main cause of chronic liver disease underlying hepatocellular carcinoma, (Dyson et al., 2014) and the fastest growing indication for liver transplantation, implicating a revision of pre- and post-transplant management (Burra et al., 2020; Haldar et al., 2019).

In the United States the prevalence of NAFLD, as assessed using ultrasound associated with transaminases increases or scores like fatty liver index/NAFLD score, reaches 19 to 46 percent in the adult population, with most biopsy-based studies reporting a prevalence of NASH of 3–5 percent (Williams et al., 2011; Vernon et al., 2011; Lazo et al., 2013). Worldwide, NAFLD has a reported prevalence of 6–35 percent (median 20 percent) depending on the classification criteria (Browning et al., 2004; Williams et al., 2011).

Estimates of prevalence of NAFLD in Asia-Pacific regions range from 5 to 30 percent, depending upon the population studied (Amarapurkar et al., 2007) while in the United States, the prevalence of NAFLD has been increasing over time, demonstrated in a comparison of three cycles of the National Health and Nutrition Examination Survey (NHANES): between 1988 and 1994, the prevalence of NAFLD was 5.5 percent, between 1999 and 2004 it was 9.8 percent, and between 2005 and 2008 reaching 11 percent, accounting for 47, 63, and 75 percent of chronic liver disease during the follow up (Younossi et al., 2011).

## Non-Alcoholic Fatty Liver Disease an Updated Pathophysiology

While NASH is considered a condition that promotes fibrosis progression, longitudinal studies have demonstrated that the liver-related prognosis of patients with NAFLD is also mostly related to the extent of liver fibrosis (Taylor et al., 2020) as observed for the other causes of chronic liver disease, such as HBV/HCV infections (D’Ambrosio et al., 2022). The pathophysiologic mechanisms involved in NAFLD have not been fully clarified yet. The most widely accepted theory holds that the main determinant of NAFLD and, possibly NASH, is insulin resistance. Many authors have proposed a model based on the necessity of an additional damage, like oxidative stress, to lead to those necro-inflammatory components typical of steatohepatitis: this is commonly referred to as second hit model. Moreover, other factors have been implied in the pathogenesis of NAFLD (e.g., antioxidant deficiencies, iron accumulation in the liver, intestinal hormones, and the changes in activity or number in gut microbioma); therefore, other authors have proposed a “multiple hits'' model hypothesis, in which multiple insults seem to act together to induce NAFLD in patient with genetic predisposition to hepatic steatosis (Buzzetti et al., 2016).

The hepatic steatosis is characterized by high accumulation of lipids (primarily triglycerides, free fatty acids (FFA), cholesterol, and ceramides) and this aberrant accumulation results in liver toxicity (Marra and Svegliati-Baroni, 2018).

Lipid accumulation in the liver has been explained involving different processes: one is the excessive importation of FFA from adipose tissue, although a diminished hepatic export of FFA, due to a lower release and production of very low-density lipoprotein (VLDL), has also been observed in NAFLD (Ipsen et al., 2018; Gaggini et al., 2013). Furthermore, in NAFLD there is also altered β-oxidation of FFA. Both elevated levels of peripheral FFA (freed from the adipose tissue where they are normally stored) and the production of FFA (as *de novo* lipogenesis process) contribute to the accumulation of hepatic and lipoprotein fat in NAFLD (Donnelly et al., 2005).

NAFLD occurs when the absorption of FFA and triglycerides from the blood and *de novo* lipogenesis exceed the oxidation rate of FFA and the export of VLDL; the impaired metabolism of lipids is also associated with the progression of NAFLD to NASH. Changes in hepatic and serum lipidomic signatures have been proposed as index of the development and progression of fatty liver disease (Alonso et al., 2019).

In NAFLD/NASH subjects, the increased FFA entry in the liver leads to mitochondrial dysfunction and lipotoxicity (Gaggini et al., 2013). Nevertheless, a clinical trial in NAFLD and NASH patients analysing electron microscopy of hepatocytes, demonstrated that significant mitochondrial abnormalities were present in patients with NASH, but not in those with hepatic steatosis, thus suggesting that peripheral insulin resistance could lead only to the development of fatty liver disease, not to inflammation as seen in steatohepatitis (Sanyal et al., 2001). Other authors suggest that mitochondrial abnormalities could be a consequence of free oxygen radical species or higher lipid peroxidation (Hruszkewycz, 1988; Chen et al., 1998).

Recent studies have shown downregulation of sirtuins in animal models and humans with NAFLD. Seven different sirtuins have been identified so far, involved in hepatic glucose and lipid metabolism, and mitochondrial function. Sirtuin 1, 3, 5, 6 were decreased in patients with NAFLD, while Sirtuin4 was increased (Wu et al., 2014). In another study (Tarantino et al., 2014b), plasma levels of sirtuin4 were low in obese patients with NAFLD. Treatment with the SIRT1 activator, SRT1720, in obese mice increased oxidative metabolism and improved insulin resistance and reduced obesity (Feige et al., 2008). Mitochondrial dysfunction in NAFLD was also related to decreased hepatic heme-oxygenase-1 (HO-1), increased NOV/CCN3, a proinflammatory adipokine, decreased peroxisome proliferator-activated receptor gamma coactivator 1-alpha (PGC-1α), a major regulator of mitochondrial function, oxygen consumption and oxidative phosphorylation (Sacerdoti et al., 2018).

## Non-Alcoholic Fatty Liver Disease Determinants and Relations With Cardiovascular Disease

Cardiovascular (CV) disease is the main cause of morbidity and mortality in patients with NAFLD (Ekstedt et al., 2015) where the CV risk factors are overexpressed. In particular higher prevalence of clinical and subclinical atherosclerosis (Fracanzani et al., 2016; Oni et al., 2013; Oni et al., 2013), coronary artery disease (Sinn et al., 2017) augmented arterial stiffness (Salvi et al., 2010), cardiac dysfunction, including heart failure and arrhythmia (Petta et al., 2015), and higher incidence of CV events, compared to the general population, are reported. (Marchesini et al., 2003; Ratziu et al., 2010). However, the studies are not all in the same direction and the NAFLD diagnosis in current routine care of 17.7 million patient appears not to be associated with acute myocardial injury or stroke risk after adjustment for CV risk factors (Alexander et al., 2019).

Trying to dissect the impact of NAFLD *per se* on CV events and death, might be questionable. However, the bidirectional relationship between NAFLD and hypertension seems to be independent of other components of the metabolic syndrome (MetS) (Oikonomou et al., 2018).

Insulin resistance is considered the main determinant of hepatic steatosis and steatohepatitis. (Chitturi et al., 2002; Willner et al., 2001; Hamaguchi et al., 2005). Also other hormons like leptin (Polyzos et al., 2015), ghrelin (Kořínková et al., 2020), adiponectin (MacHado et al., 2012), resistin (Polyzos et al., 2016), incretin like GLP-1 (Svegliati-Baroni et al., 2020) are described to be linked with the NAFLD genesis.

NAFLD patients are often obese and/or affected by type 2 diabetes mellitus, two conditions associated with peripheral insulin resistance. Nevertheless, insulin resistance has also been observed in non-obese NASH patients and in those who have normal glycemic levels, thus suggesting a strong association between insulin resistance and lipid accumulation (Chitturi et al., 2002; Marchesini et al., 2001; Marchesini et al., 2003; Hae et al., 2004). Subjects with NAFLD and glucose intolerance seem to be significantly more insulin resistant than those with glucose intolerance, but without NAFLD (Facchini et al., 2002; Kelley et al., 2003).

Visceral fat accumulation is considered an independent risk factor in NASH patients, as it has been suggested that a higher visceral fat level in these patients leads to higher liver fibrosis and inflammation: this could be linked to proinflammatory cytokines activity, like interleukin-6 (IL-6) (van der Poorten et al., 2008; Wieckowska et al., 2008), or activation of tumor necrosis factor alpha-converting enzyme (TACE), as observed in the experimental animal model (Fiorentino et al., 2010; de Meijer et al., 2011). Once established, insulin resistance leads to important alterations in the metabolism of lipids, such as increase in the absorption of FFA by the liver, peripheral lipolysis and the synthesis of triglycerides (Kral et al., 1977). The result is the preferential shift from carbohydrates to β-oxidation of FFA in the liver, an event that has been demonstrated in patients with insulin resistance (Sanyal et al., 2001; Alonso et al., 2019).

Several studies have investigated the possible genetic polymorphisms present in patients with NAFLD/NASH and the results obtained have suggested a certain role of IL-6, adiponutrin apolipoprotein C3, and the peroxisome proliferator-activated receptor gamma coactivator 1-alpha (PPARGC1A) (Carulli et al., 2009; Rotman et al., 2010; Petersen et al., 2010), (Sookoian et al., 2010b; Domenici et al., 2013). Recently another cytokine proposed in atherosclerotic NAFLD patients was IL-17A (Tarantino et al., 2014a), the same cytokine that exerts pro-aggregant effects (Maione et al., 2011). However no consistent data are still available.

It has been suggested that the phenotype of manifestations of NAFLD and the progression of the disease are the result of complex interactions between the environment and the genetic pool of the subject. Some studies highlight a strong heritability of lipid content in the liver (Dongiovanni et al., 2013) and familial and twin studies support the hypothesis of a heritable effect of NAFLD (Willner et al., 2001; Struben et al., 2000; Schwimmer et al., 2009).

There are many genes associated with insulin signaling and lipid metabolism, which are involved in the development of NAFLD, and it is not our aim to give them a full discussion here. However it is important to list at least the genetic polymorphisms of greatest interest in this pathology. Notably, patatin-like phospholipase domain-containing protein 3 (PNPLA3) I148M variant is the commonest inherited determinant of NAFLD as it is associated with progression of NAFLD, NASH, and NAFLD-related HCC (Dongiovanni et al., 2015; Eslam et al., 2018; Seko et al., 2018; Trépo and Valenti, 2020). The PNPLA3 isoleucine to methionine substitution at position 148 (I148M) induces a loss-of-function in the enzymatic activity, resulting in accumulation of triacylglycerol in the liver: in fact PNPLA3 protein catalyzes hydrolysis of glycerolipids, such as the triacylglycerol; the I148M mutation results in a loss-of-function, thus contributing to the entrapment of triglycerides in lipid droplets of hepatocytes and hepatic stellate cells. (Huang et al., 2011; Pingitore et al., 2014; Eslam et al., 2018). Moreover, homozygosity for the PNPLA3 I148I variant is associated to a 10-fold increased risk of HCC related to NAFLD in the general European population (Liu et al., 2014; Eslam et al., 2018; Choudhary and Duseja, 2021), while the PNPLA3 S453I polymorfism plays a protective role (Choudhary and Duseja, 2021).

The Transmembrane 6 superfamily member 2 (TM6SF2) gene E167K variant promotes a reduction in VLDL secretion from the liver, inducing higher triglycerides levels in the liver and a lower capacity of LDL secretion from hepatocytes, while patients with TM6SF2 E167K polimorfism show a reduced cardiovascular risk (Dongiovanni et al., 2015; Eslam et al., 2018; Choudhary and Duseja, 2021)**.** A well-known genetic polymorphism studied in NAFLD subjects is the Membrane bound O-acyltransferase domain-containing 7 (MBOAT7) downregulation: MBOAT7 is a gene implicated in phosphatidylinositol (and other phospholipids) remodeling *via* the incorporation of arachidonic acid and other unsaturated fatty acids into lyso-phospholipids. The common genetic variant leads to a downregulation of MBOAT7 activity and consequantely to accumulation of lyso-phosphatidyl-inositol in hepatocytes; this in the end leads to a higher sinthesis of triglycerids in the liver and NAFLD. (Eslam et al., 2018; Donati et al., 2017; Trépo and Valenti, 2020; Choudhary and Duseja, 2021).

Glucokinase regulator (GCKR) controls the glucose inflow in hepatocytes thus regulating the *de novo* lipogenesis in the liver; the GCKR P446L is a missens variant that causes a protein loss of function, in the end resulting in a constitutively active glucose inflow in the hepatocytes (Eslam et al., 2018; Trépo and Valenti, 2020), (Choudhary & Duseja, 2021).

The protein phosphatase 1 regulatory subunit 3B (PPP1R3B) encodes for a protein involved in glycogen synthesis. PPP1R3B rs4841132 variant has been suggested to protect against hepatic fat accumulation and liver fibrosis in NAFLD subjects (but not in the general population): this variant increases the lipid oxidation and downregulates some lipid metabolism and inflammation pathways (Dongiovanni et al., 2018; Stender et al., 2018)**.**

Interferon lambda 4 (IFNL4) rs368234815 variant is associated to higher inflammation and fibrosis in the liver; Mer T kinase (MERTK) variants could alter the hepatic inflammation and fibrosis *via* the modulation of phagocytes and hepatic stellate cells activity, and a specific variant (MERTK rs4374383) exerts a protective role, reducing the MERTK expression in the liver (Trépo and Valenti, 2020; Choudhary and Duseja, 2021).

HSD17B13 gene encodes for an enzyme that concentrates lipid droplets in hepatocytes: loss-of-function variants in HSD17B13 result in higher protection against liver inflammation, cirrhosis, and HCC (Trépo & Valenti, 2020; Choudhary and Duseja, 2021).

Another important pathogenetic factor is represented by the accumulation of iron, which can contribute to the development of NASH and promote oxidative stress, thus producing free oxygen species which lead to liver damage and fibrosis, and in the end NASH (Chitturi and George, 2003).

Nevertheless, studying hemochromatosis gene (HFE) mutations, no significant role in the development of insulin resistance-associated liver siderosis was seen, apart from compound heterozygosity (Guillygomarc’h et al., 2000).

Unexplained hepatic iron overload is frequently associated with the insulin-resistance syndrome irrespective of liver damage. This insulin-associated iron overload is characterized by a mild to moderate iron excess with hyperferritinemia and normal to mildly increased transferrin saturation (Mendler et al., 1999).

The global number and complex activities of intestinal microbes, referred as “gut microbiota”, affect hepatic carbohydrate and lipid metabolism, and can alter the balance between proinflammatory and anti-inflammatory mechanisms happening in the liver, so that gut microbiota is involved in NAFLD development and eventually in its progression to NASH, promoting lipotoxicity in the liver and influencing pathogenesis of NAFLD/NASH with multiple mechanisms, including translocation of dysbiotic bacteria and their derived products to the liver through a disrupted or more permeable gut barrier (Kolodziejczyk et al., 2019; Safari and Gérard, 2019). Gut microbiota may also contribute to liver damage by means of endotoxin production, which leads to gut barrier alterations and proinflammatory enhancement, thus promoting worsening of NAFLD/NASH (Kolodziejczyk et al., 2019; Wesolowski et al., 2017), (Verdam et al., 2011; Hu et al., 2020), and *via* bile salts deconjugation, and inactivation of hepatic lipotropic molecules (such as choline) (Hu et al., 2020).

The production of endogenous alcohol and acetaldehyde (the so-called auto-brewery syndrome) is another suggested mechanism (Cope et al., 2000): colonic bacteria and yeast have a high metabolic ability in producing both ethanol and acetaldehyde, and they can oxidize ethanol to high levels of acetaldehyde. Acetaldehyde, then, can be easily absorbed into the portal blood stream and begin histologic changes like those seen in NAFLD (Salaspuro, 1996).

Other factors and mechanisms have been studied relating to the pathogenesis of NAFLD. The cholesterol intake with diet has been proposed as an independent factor in developing of NASH (Wouters et al., 2008). It has been suggested that even obstructive sleep apnea syndrome (OSAS) could have a role in inducing inflammation in NAFLD (Zamora-Valdés and Méndez-Sánchez, 2007). Another proposed pathogenetic factor in NAFLD is thyroid hormone receptor-β. Altered signaling of thyroid hormones may result in altered lipid metabolism and may also have a role in the development of NAFLD (Ritter et al., 2020; Saponaro et al., 2020; Attia et al., 2021).

## The Role of Platelets in Non-Alcoholic Fatty Liver Disease/Non Alcoholic Steatohepatitis

Platelets are involved in different models of liver damage (Fujita et al., 2008; Lang et al., 2008; Iannacone et al., 2005; Sitia et al., 2012; Iannacone et al., 2005).

Apart from the well-defined interaction between CV risk factors and NAFLD/NASH, a pro-thrombotic condition may derive from altered endothelial and vascular function and platelet activation and interaction with blood and liver cells.

In most of the cases, NASH develops in the context of a metabolic syndrome, which is a pro-thrombotic and pro-atherogenic condition (McCracken et al., 2018). Taking this into account, today it is still debated whether NASH contributes to an enhanced risk of CV disease per se, but on the converse, it has been demonstrated that vascular lesions in the liver contribute to the pathogenesis of NASH (Francque et al., 2012; Lefere et al., 2019; Adori et al., 2021). Many different mechanisms may explain how NASH could contribute to vascular disease, for example, by increasing the production of pro-thrombotic factors by the liver, like plasminogen activator inhibitor-1 (PAI-1), which has been shown to have higher activity in patients with metabolic syndrome (Mertens and Van Gaal, 2006). Recently, it has also been reported that in some of the patients with non-cirrhotic NASH, the liver presents some lesions described as “atypical”, for example, porto-sinusoidal venous disease-like (PSVD-like) lesions (Brunt et al., 2021). Francque et al. have hypothesized that increased intrahepatic vascular resistance contributes to NALFD progression *via* intralobular hypoxia and local ischemia, (Francque et al., 2012; Van der Graaff et al., 2018; Pasarín et al., 2012).

NASH represents the result of the effect of a chronic inflammatory state on the liver. The inflammatory state is mostly due to metabolic disbalance leading to metabolic syndrome, obesity, insulin resistance and diabetes (type 2). Lipid species cause inflammation and activation of both infiltrating and resident immune cells. How are platelets involved in this process? Platelets are involved in pathological processes such as chronic inflammation and atherothrombosis and possibly fibrogenesis (Matsushita et al., 2020).

Platelets contain granules that are released in response to activatory stimuli (platelet release reaction). Alpha and delta (dense) granules may release in the microenvironment the proaggregatory factors ADP, serotonin and thrombin (the amplificatory process) along with appreciable amounts of inflammatory cytokines, chemokines and growth factors such as platelet-derived growth factor (PDGF), endothelial growth factor (EGF), insulin-like growth factor 1 (IGF-1), transforming growth factor beta (TGFβ), tumor necrosis factor alpha (TNFα), interleukin-6 (IL-6), chemokine ligand 4 (CXCL4), vascular endothelial growth factor A (VEGF-A), hepatocyte growth factor (HGF), fibroblast growth factor (FGF). (Heijnen and van der Sluijs, 2015; Taus et al., 2019). Platelets are able to store and even synthesize interleukin (IL)-1, plasminogen activator inhibitor-1 (PAI-1) and tissue factor (TF). Therefore, platelets express receptors and activities that are not only involved in the generation of platelet aggregates and, as the leptin receptor, thrombus formation, but also inflammation (Bodary et al., 2002; van der Meijden and Heemskerk, 2019).

Platelets release factors that change gene expression in endothelial cells, leukocytes, stromal cells and fibroblasts thus directly participating to inflammation (van der Meijden and Heemskerk, 2019). Platelets and platelet-derived microparticles (PMPs) also deliver mRNA and miRNA that are incorporated in target cells changing their phenotype (Xia et al., 2018).

In patients with metabolic syndrome, increased platelet count, platelet distribution width (PDW), mean platelet volume (MPV) values, and platelets/lymphocyte ratio have been associated with hyperleptinemia and hypoadiponectinemia (Abdel-Moneim et al., 2019). In obese subjects, platelets show increased aggregability and activation (Beavers et al., 2015). Increased MPV has been considered as a marker of *in vivo* platelet activation (Coban et al., 2005). In NAFLD patients, lower platelet count and higher MPV have been observed, nonetheless other Authors did not confirm these alterations (Tuzer et al., 2021).

In overweight and obese insulin resistant subjects, plasma concentrations of P-selectin are increased, and decrease after weight loss (Russo et al., 2010). In obese mice the genetic or antibody mediated disruption of CD40L signalling, which is related to platelet activation and cell-cell communication, improves adipose tissue inflammation and metabolic disorders in insulin resistance (Poggi et al., 2011). Activated platelets lose 0.1–1-μm fragments called PMPs, which express functional receptors from platelet membranes, like the procoagulant phosphatidylserine. PMPs can induce thrombosis and inflammation (Burnouf et al., 2014). PMPs regulate 1) expression of cyclooxygenase-2 (COX-2) and prostacyclin (PGI2) in endothelial cells (Barry et al., 1997), 2) monocytes and endothelial cells interaction by means of the expression of ICAM-1 (Smith et al., 1989), 3) aggregation and accumulation of neutrophils through the expression of P-Selectin and IL-1 (Forlow et al., 2000), 4) production of pro-inflammatory molecules: CD40L, IL-1, IL-6 and TNF-α (Cloutier et al., 2013), and 5) C reactive protein (CRP) production, enhancing the local inflammatory response *via* activation of the classic complement pathway (Braig et al., 2017). The inflammatory role of PMPs has been observed in several pathologies, particularly in chronic inflammation, typically connected to tissue damage, such as in cardiovascular diseases, rheumatoid arthritis, anti-phospholipid antibody syndrome, mellitus diabetes (Beyer and Pisetsky, 2010).

In obese non-diabetic subjects, elevated circulating number of PMPs positively correlate with BMI and waist circumference. Weight reduction reduces the release of PMPs (Murakami et al., 2007). Interestingly, another study has recently shown that PMPs from obese subjects were not altered in number, if compared with non-obese subjects, but were heterogeneous in size and distribution, with different levels of proteins relevant to thrombosis and tumorigenesis (Grande et al., 2019).

In patients with insulin resistance, the inhibitory effects of insulin on platelets are impaired (Simon et al., 2019), due to the abnormal adipokine content (Gerrits et al., 2012). In particular, the adipokines resistin, leptin, PAI-1 and retinol binding protein 4 (RBP4) induce insulin resistance in megakaryocytes by interfering with IRS-1 expression with a negative impact on insulin signalling in platelets (Gerrits et al., 2012). Liraglutide, an incretin hormone glucagon-like peptide 1 (GLP-1) analogue, has been shown to inhibit platelet activation in animal models (Cameron-Vendrig et al., 2016) and human platelets (Barale et al., 2017).

Hyperglycemia is a causal factor of platelet hyperreactivity, as indicated by enhanced aggregation, increased fibrinogen binding, and thromboxane A2 (TXA_2_) production (Davì et al., 1990; Tang et al., 2011). Platelets from diabetic patients undergo spontaneous aggregation (Matsuno et al., 2005) as well as increased adhesion and aggregation in response to agonists (Watala, 2005).

Platelets from obese, insulin-resistant individuals are characterized by impaired response to nitric oxide (NO) and altered downstream cGMP/cGMP-dependent protein kinase (PKG) signalling system. Similarly, the inhibitory activity of prostacylin (PGI2) towards platelet activation and the engagement of the cAMP/cAMP-dependent protein-kinase (PKA) pathways are impaired (Anfossi et al., 2004). This is associated with enhanced activatory signals including increase in free intracellular calcium and the expression of platelet activation markers including the release of PMPs, potential predictors of cardiovascular risk (Tang et al., 2011; Santilli et al., 2016). Consistently with these observations, the activity of antiplatelet drugs was found blunted in diabetic patients (Braunwald et al., 2008).

Increased oxidative stress, derived from hyperglycemia and platelet activation, potentiates cytosolic phospholipase A_2_ signalling, which catalyses arachidonic acid release and TXA_2_ generation. Activation of the aldose reductase pathway is implicated in oxidative stress-induced TXA_2_ biosynthesis amplified by exposure to collagen, indicating that when the vascular endothelium is damaged thromboembolic events are promoted (Tang et al., 2011). Increased TXA_2_-dependent platelet activation is mediated by PKC/p38MAPK signals and also associated with enhanced CD40L release (Tang et al., 2011; Santilli et al., 2006). High glucose concentrations are also determinants of loss of function and damage to mitochondria in platelets, mitochondrial membrane potential dissipation, cytochrome c release, caspase-3 activation, leading in subgroups of platelets to apoptosis (Tang et al., 2014). Platelets from diabetic individuals also show reduced sensitivity to the antiaggregatory effects of insulin, NO, and PGI_2_ (Anfossi et al., 2006). Since antiplatelet effects are related to increased platelet NO synthesis, sensitivity to NO signalling may account, at least partially, for less protective aspirin effects against thrombotic events in type 2 diabetes mellitus (Russo et al., 2012).

Hyperaggregability has been observed in platelets from subjects with hypercholesterolemia, along with increased fibrinogen binding and surface expression of P-selectin, increased generation of TXA_2_ and superoxide anion. Plasma from the same patients contains increased concentrations of platelet activation markers, including soluble CD-40L, soluble P-selectin, PF-4 and thromboglobulin (Akkerman, 2008; Barale et al., 2018; Barale et al., 2020). Triglycerides-rich particles have been shown to directly activate platelets (Yamazaki et al., 2005).

Platelet aggregation in response to various agonists, including collagen, ADP, arachidonic acid and TXA_2_ is increased in obese patients (Bordeaux et al., 2010; Barrachina et al., 2019). The adipokine leptin provides a potential link between platelets, obesity and NAFLD. Leptin correlates with the severity of NAFLD or NASH and promotes arterial thrombosis in a platelet leptin receptor-dependent manner (Bodary et al., 2002; Rotundo et al., 2018). Leptin enhances ADP-induced platelet aggregation at clinically relevant concentrations (Corsonello et al., 2002; A. Corsonello et al., 2003).

TXA_2_ release as well as hepatic TXA_2_ receptor (TP) expression are upregulated in NAFLD (W. Wang et al., 2018). NAFLD further results in a hypercoagulatory state with an increased thrombotic risk due to elevated levels of vWF and plasminogen activator inhibitor type I (PAI-1). The activated coagulation cascade in NAFLD leads to thrombin generation, which not only cleaves fibrinogen into fibrin, but is also a strong platelet activator *via* proteinase activated receptor 1–4 (PAR1–4) signalling, leading to platelet hyperreactivity. While experimental models confirm thrombin generation in NAFLD clinical evidence, is lacking (Kopec et al., 2014).

Patients and mice with NAFLD have increased blood levels of molecules present in granules from platelets. Thrombospondin (TSP-1), present in platelets (Mussbacher et al., 2021), but synthesized also by hepatic stellate cells (HSC), Kupffer cells, endothelial cells, and adipocytes (Bai et al., 2020), exerts a beneficial effect on NAFLD due to inhibition of genes promoting lipid production (Bai et al., 2020).

One mechanism of interaction between platelets and leukocytes is through CD40L. CD40L belongs to the TNF superfamily, and is increased in NAFLD platelet surface, signalling leukocytes expressing CD40 (Sookoian et al., 2010a). It has been shown that inhibition of this mechanism by antibodies against CD40L or genetic manipulation, decreases the effects of diet on steatosis, adipose tissue accumulation and insulin resistance (Poggi et al., 2011), acting on hepatic very low density lipoprotein (VLDL) secretion and genes regulating lipid balance (Villeneuve et al., 2010).

Glycosaminoglycans and CXCR3, a chemokine receptor, bind to CXCL4, a protein secreted by platelets (Lasagni et al., 2003). In experimental NASH, CXCR3 increases the amount of lipids, and causes endoplasmic reticulum stress (Zhang et al., 2016). Another important platelet-derived mediator is serotinin (Starlinger et al., 2021).

Platelets may control gene expression in hepatocyte, with possible implications in liver diseases, also by delivering genetic information to the target cells. Direct transfer of mRNA from platelets to hepatocytes has been demonstrated using HepG2 cells, which internalised platelets. Platelets internalisation has been observed also following a partial hepatectomy in mice and is associated with hepatocyte proliferation. Enzymatic removal of platelet-derived RNA blunts hepatocyte proliferation (Kirschbaum et al., 2015). Transfer of miRNA from platelets *via* platelet-derived microparticles to hepatocytes has also been demonstrated. PMP carrying miR-25-3p promoted hepatocyte proliferation modifying gene expression (Xu et al., 2020).

An etiologic role of platelets in development of NASH has been suggested by demonstrating that they can be found in the steatotic liver before the presence of leukocytes, thus hypothesizing that immune cells are recruited by platelets. The hypothesis is confirmed by the effect of reducing platelet number or antiplatelet agents on inflammation in experimental animals (Malehmir et al., 2019). Mediators of platelets inflammatory effect are granules content and activation of leukocytes through the GP1b receptor, as demonstrated by the efficacy of aspirin in decreasing the development of NASH and fibrosis. (Simon et al., 2019).

As concerns this last point, i.e., fibrosis, it is well known that platelets can interact with hepatic stellate cells (HSC) through mediators with both pro- and anti-fibrotic effects (Kurokawa and Ohkohchi, 2017). Adenine nucleotides and HGF from platelets granules have antifibrotic effects (N. Ikeda et al., 2012; Kodama et al., 2010; Kim et al., 2005) and these beneficial effects are confirmed by the reduction of liver fibrosis following treatment with platelet-rich plasma (PRP) (Salem et al., 2018; Takahashi et al., 2013).

The profibrotic effects of activated platelets (Yoshida et al., 2014) are due to hepatic microthrombosis (Zaldivar et al., 2010), TGF (Mahmoud et al., 2019), PDGF-B (Kinnman et al., 2003), vWF (Joshi et al., 2017), platelet-derived S1P signalling (H. Ikeda et al., 2000; King et al., 2017; Rohrbach et al., 2017) which activate HSC to increase collagen secretion (Ghafoory et al., 2018), proliferate, migrate and become myofibroblasts, (Kim et al., 2005; Yoshida et al., 2014; H. Ikeda et al., 2000; Borkham-Kamphorst et al., 2007). Also serotonin has profibrotic activity, through receptors that increase TGF, collagen, and other factors (Ruddell et al., 2006).

The real role of platelets in increasing liver fibrosis is underlined by the positive effect of aspirin (Poujol-Robert et al., 2014; Jiang et al., 2016), documented also in patients (Jiang et al., 2016), and other inhibitors of platelet function, antiplatelet clopidogrel and anticoagulant dabigatran, which decrease TGF, smooth muscle actin, and collagen (Mahmoud et al., 2019).

Regarding immune response, both innate and adaptive, platelets play a very important role in its stimulation. Activated platelets attract immune cells and modulate inflammation through the expression of specific receptors and release of chemokines and cytokines (Semple et al., 2011) in the liver and spleen (Maini and Schurich, 2012), but not through aggregation. Activation of Kuppfer cells is also dependent on the presence of platelets (Pereboom et al., 2008).

According to Malehmir et al., platelets are involved only in pathophysiology of NASH, while they do not play any role in steatosis (Malehmir et al., 2019). In experimental NASH there is an increase in platelet numbers, aggregation and activation in the liver, not associated with an increase in peripheral number. In this experimental condition, activated partial thromboplastin time (aPTT) is also significantly reduced. These platelets alterations are reduced by aspirin-clopidogrel, together with a reduction of ALT, AST, liver/body weight ratio, liver triglycerides, serum total cholesterol, LDL and HDL cholesterol, and an improvement in glucose tolerance. These effects are associated with a reduction of platelets activation, as demonstrated by the decrease in the response to agonists. Furthermore, also immune cell infiltration is reduced (Malehmir et al., 2019). In the pilot study of Malehmir et al., in patients with NAFLD, treatment with antiplatelet drugs for 6 months caused a significant decrease in liver volume and liver fat mass (Malehmir et al., 2019).

In patients with NASH and in mice with choline deficient high fat diet (CDHFD) induced NASH there is increased hepatic infiltration of CD3^+^CD8^+^ T cells, CD11b + MHCII + myeloid cells and Ly6G + granulocytes (Wolf et al., 2014). Treatment with aspirin-clopidogrel causes a reduction of immune cell infiltration (Malehmir et al., 2019). Aspirin-clopidogrel significantly reduces CD11b + F4/80hi Kupffer cells, Kupffer cell activation, inflammatory myeloid cell hepatic infiltration (He and Karin, 2011).

The demonstration that the improvement of NASH with the combined treatment with aspirin-clopidogrel is not COX-dependent comes from the evidence that in experimental NASH, sulindac, only a COX-inhibitor, does not modify obesity, liver/body weight ratio, hepatic triglycerides and glucose tolerance, liver damage (Malehmir et al., 2019).

Considering the role of platelets receptors in NASH, it has been shown that the GPIIb subunit of the platelet fibrinogen receptor GPIIb/IIIa is not involved, confirming that platelets aggregation is not responsible for NASH (Malehmir et al., 2019), as well as platelet integrin α2β3 binding motif of fibrinogen (Kopec et al., 2017). On the contrary, attachment and activation of platelets is associated with platelet-derived GPIbα (Haemmerle et al., 2018), as confirmed by the improvement of steatosis, hepatic injury, triglycerides content, fibrosis and leukocytes infiltration when the major ligand binding domain of GPIbα is blocked, reducing the interaction with Kupffer cells. Furthermore, cytokines and chemokines produced by Kupffer cells are decreased in the liver by anti-GPIbα antibody treatment (Malehmir et al., 2019).

Development of diet induced NASH was not affectd by deletion of P-selectin (Selp–/–), (Subramaniam et al., 1996), von-Willebrand-factor (vWF–/–) (Blenner et al., 2014) or Mac-1 (Mac-1−/−) (Y. Wang et al., 2017), the major platelet adhesion receptors (Malehmir et al., 2019). Inflammation of liver microvasculature and recruitment of immune cells also do depend on selectins (Wong et al., 1997).

**FIGURE 1:**
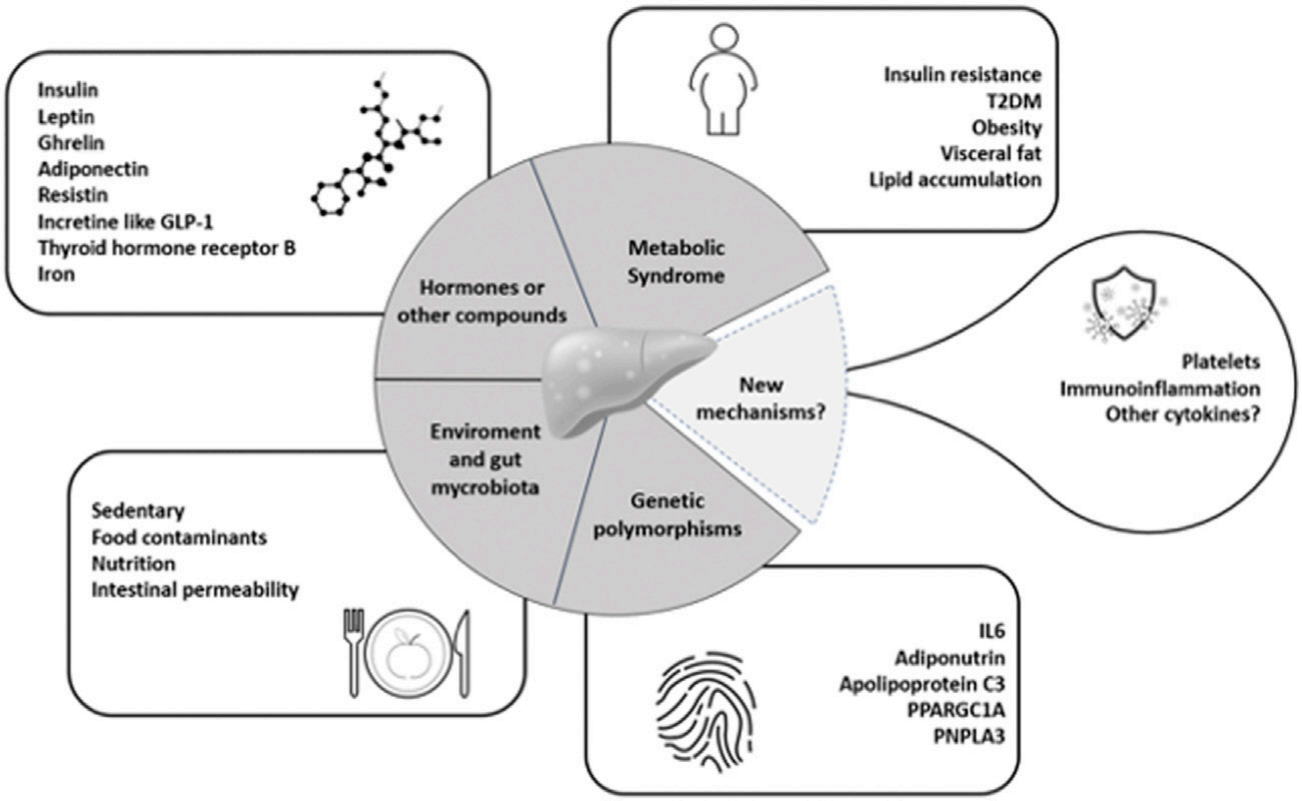
Main determinants of NAFLD/NASH

Recently, the contribution of platelets to liver inflammation was confirmed by immunohistochemical staining on liver biopsies showing accumulation of platelet and neutrophil extracellular traps (NET) in liver, with a correlation with NAFLD activity score. Circulating platelets from patients with NAFLD were shown to have significant increase of inflammatory transcripts, while leukocytes did not (Miele et al., 2021) (Figure 1).

## Antiplatelet Agents in Non-Alcoholic Fatty Liver Disease/Non Alcoholic Steatohepatitis

Few studies have addressed the effect of antiplatelet therapy in NAFLD/NASH, as previously anticipated.

In 2008, Fujita et al. have shown that three antiplatelet drugs, aspirin, ticlopidine and cilostazol, can improve liver steatosis, inflammation and fibrosis in experimental dietary NAFLD. These drugs reduce oxidative stress, that activates mitogen-activated protein kinase, and platelet-derived growth factor *via* intercepting signal transduction from Akt to c-Raf (Fujita et al., 2008). Cilostazol was also shown to reduce fibrogenesis induced by CCl4, suppressing stellate cells activation. (Saito et al., 2014).

In 2011, Ibrahim et al. have shown that NO-aspirin, but not aspirin, can prevent the development of cholesterol-induced NAFLD, by decreasing iNOS and COX-2 activity (Ibrahim et al., 2011).

The first suggestion of a clinically beneficial role of aspirin was published in 2014 (Shen et al., 2014). In a cross-sectional analysis of data from 11,416 adults, aspirin (taken ≥15 times per month) was inversely associated with the presence of NAFLD, primarily among men and older patients.

A role of aspirin in development of fibrosis was suggested by (Jiang et al., 2016). In a cross-sectional analysis in 1856 patients with suspected chronic liver disease, they showed that aspirin was associated with significantly lower indices of liver fibrosis.

The role of platelet inhibition with aspirin is confirmed by (Simon et al., 2019). In a prospective study of 361 adults with biopsy-confirmed NAFLD they showed that daily aspirin is associated with less severe histologic features of NAFLD and NASH, and lower risk to progress to advanced fibrosis.

Aspirin inhibits lipid biosynthesis and inflammation and increase catabolism through the activation of the PPARδ-AMPK-PGC-1α pathway. Furthermore, aspirin may modulate the mannose receptor and CCR2 in macrophages. (Han et al., 2020). The antifibrotic role of aspirin was shown also in transplanted patients with recurrence of hepatitis C (Poujol-Robert et al., 2014) One last experimental study in Guinea pigs did not find any effect of aspirin on steatosis, NASH, or hepatic fibrosis (Ipsen et al., 2021).

An improvement of the oxidative, inflammatory and fibrosis markers, TGF, smooth muscle actin, and collagen, as well as histopathological changes with two different antithrombotic drugs, anticoagulant dabigatran and antiplatelet clopidogrel, in rats given CCl4, was also shown (Mahmoud et al., 2019).

The most striking evidence of the benefit of platelet inhibition is shown by Malehmir et al. (Malehmir et al., 2019). They show how the association aspirin-clopidogrel lowers intrahepatic platelet numbers, reduces platelet aggregation and activation. In patients with NAFLD aspirin-clopidogrel reduced liver volume, liver fat mass, CD3^+^ T cell infiltration, ALT, AST, liver/body weight ratio, platelet numbers and aggregation state. Aspirin-clopidogrel significantly improved glucose tolerance, reduced liver triglycerides and attenuated serum total cholesterol, LDL and HDL cholesterol. In mice with CDHFD treated with aspirin-clopidogrel, integrin αIIbβ3 activation and P-selectin exposure were reduced, as well as the response of circulating platelets to agonists, a proof of reduction in platelet activation. As a consequence, leukocytes infiltration, total number, effector differentiation (CD8^+^CD62L–CD44^+^CD69^+^) and proportion of CD4+/CD8+ and NKT cells, CD11b^+^F4/80hi, Kupffer cells, Kupffer cell activation, inflammatory myeloid cell infiltration were reduced. Thus, aspirin-clopidogrel prevented NASH and reduced NASH-related increase of platelet interaction with the liver endothelium, T cells and innate immune cells.

Finally, antiplatelet treatment seems to be effective only in the liver, affecting interactions of GPIbα+ platelets with Kupffer cells, in mouse and human NASH (Malehmir et al., 2019).

## Conclusion

Scientific evidence supports the hypothesis that platelets are implicated in the pathophysiology of NAFLD/NASH, mostly by exerting proinflammatory and profibrotic activities, rather than exerting their thrombogenic activities. The recently discovered interaction of platelets with liver cells and the immune system introduces new models of inflammation and fibrogenesis in the setting of chronic liver diseases, anticipating the potential efficacy of antiplatelet agents to prevent the progression of NAFLD towards NASH and the eventual liver cancer. Further research is required to identify detailed mechanisms and potential specific target of pharmacological intervention. Clinical trials in selected patients, who may benefit of antiplatelet intervention, are warranted.
